# 2332. COVID-19 Primary Series and Booster Vaccination and Potential for Immune Imprinting

**DOI:** 10.1093/ofid/ofad500.1954

**Published:** 2023-11-27

**Authors:** Hiam Chemaitelly, Houssein H Ayoub, Laith J Abu-Raddad

**Affiliations:** Weill Cornell Medicine - Qatar, Doha, Ad Dawhah, Qatar; Weill Cornell Medicine - Qatar, Doha, Ad Dawhah, Qatar; Weill Cornell Medicine-Qatar, Doha, Ar Rayyan, Qatar

## Abstract

**Background:**

Epidemiological evidence for immune imprinting was investigated in immune histories related to vaccination in Qatar from onset of the omicron wave, on December 19, 2021, through September 15, 2022.

**Methods:**

Matched, retrospective, cohort studies were conducted to investigate differences in incidence of SARS-CoV-2 reinfection in the national cohort of persons who had a primary omicron infection, but different vaccination histories. History of primary-series (two-dose) vaccination was compared to that of no vaccination, history of booster (three-dose) vaccination was compared to that of two-dose vaccination, and history of booster vaccination was compared to that of no vaccination. Associations were estimated using Cox proportional-hazards regression models.

**Results:**

The adjusted hazard ratio for incidence of reinfection, including also adjustment for differences in testing rate, in the two-dose cohort relative to that in the unvaccinated cohort was 0.43 (95% CI: 0.39-0.49). The adjusted hazard ratio comparing incidence of reinfection in the three-dose cohort to that in the two-dose cohort was 1.47 (95% CI: 1.23-1.76). The adjusted hazard ratio comparing incidence of reinfection in the three-dose cohort to that in the unvaccinated cohort was 0.57 (95% CI: 0.48-0.68). All adjusted hazard ratios appeared stable over 6 months of follow-up. Divergence in cumulative incidence curves in all comparisons increased markedly when incidence was dominated by BA.4/BA.5 and BA.2.75*. No reinfection in any cohort progressed to severe, critical, or fatal COVID-19.

**Conclusion:**

History of primary-series vaccination enhanced immune protection against omicron reinfection, but history of booster vaccination compromised protection against omicron reinfection. These findings do not undermine the short-term public health utility of booster vaccination.

**Disclosures:**

**All Authors**: No reported disclosures

